# The Preclinical Research Progress of Stem Cells Therapy in Parkinson's Disease

**DOI:** 10.1155/2016/5683097

**Published:** 2016-06-09

**Authors:** Jun Zhang, Xianyun Wang, Jing Li, Rui Huang, Xuerui Yu, Ci Dong, Pujuan Liu, Fan Zhang, Jie Hu, Yixin Qi, Jing Zhang, Quanhai Li, Baoyong Yan

**Affiliations:** ^1^Cell Therapy Laboratory, The First Hospital of Hebei Medical University, Shijiazhuang, Hebei 050031, China; ^2^Department of Immunology, Basic Medical College, Hebei Medical University, Shijiazhuang, Hebei 050017, China; ^3^Department of Paediatrics, The First Hospital of Hebei Medical University, Shijiazhuang, Hebei 050031, China; ^4^Department of General Surgery, The First Hospital of Hebei Medical University, Shijiazhuang, Hebei 050031, China; ^5^Department of Neurology, The First Hospital of Hebei Medical University, Shijiazhuang, Hebei 050031, China; ^6^Department of Cardiac Surgery, The First Hospital of Hebei Medical University, Shijiazhuang, Hebei 050031, China; ^7^School of Nursing, Hebei Medical University, Shijiazhuang, Hebei, China; ^8^Department of Breast Center, The Fourth Hospital of Hebei Medical University, Shijiazhuang, Hebei, China

## Abstract

Parkinson's disease (PD) is a type of degenerative disorder of the basal ganglia, causing tremor at rest, muscle rigidity hypokinesia, and dementia. The effectiveness of drug treatments gradually diminishes because the conversion to dopamine within the brain is increasingly disrupted by the progressive degeneration of the dopaminergic terminals. After long-term treatment, most patients with PD suffer from disability that cannot be satisfactorily controlled. To solve these issues, stem cells have recently been used for cell therapy of PD. In this review, the characteristics of different stem cells and their therapeutic effects on PD treatment will be discussed.

## 1. Introduction

Parkinson's disease (PD) is the second most common neurodegenerative disorder in people over the age of sixty. Aging is the major contributing factor for increased risk of developing PD. With the aging of the population worldwide, the frequency of PD is expected to increase dramatically in the coming decades. PD is a chronic and progressive movement disorder, mainly characterized by the degeneration of dopaminergic (DA) neurons in the substantia nigra pars compacta coupled with intracytoplasmic proteinaceous inclusions known as Lewy bodies [1–3]. Clinical symptoms include resting tremor, rigidity, bradykinesia or slowness, gait disturbance, and postural instability. Current clinical treatments include the oral administration of levodopa (L-dopa) and other dopamine receptor agonists and deep-brain stimulation in the subthalamic nucleus. The oral administration of L-dopa provides benefit to most PD patients, resulting in the improvement of daily activities. However, long-term treatment with L-dopa is associated with many adverse events, including motor fluctuations, dyskinesias, and neuropsychiatric complications [4, 5]. So far most medical and surgical interferences fail to stop the progression of the disease. Particularly, some nondopaminergic features of the disease, such as freezing, falling, and dementia, lead to disabilities of many PD patients [6]. Clinical studies have been focused on understanding the etiology and pathogenesis of PD in the hope of developing more effective therapies that will slow or halt the disease progression.

Alternatively, stem cell therapy holds great promise in PD treatment. Stem cells are undifferentiated cells with the ability to self-renew and to differentiate into distinct types of functional cells. Stem cells can be used to generate DA neurons to replace the diseased neurons in PD patients after transplantation and engraftment. A variety of stem cells, including embryonic stem cells (ESCs), mesenchymal stem cells (MSCs), and neural stem cells (NSCs) have been reported [7–9]. In this review paper, the stem cell sources for PD therapy and their efficacy will be discussed.

## 2. Embryonic Stem Cells (ESCs)

ESCs are capable of differentiation into all body cell types. ESCs have been induced to differentiate into NSCs or precursor cells, and further induced into DA neurons [10, 11]. Two approaches have been established for neuronal differentiation of human ESCs embryoid body intermediates route and coculture method [12, 13]. Transplantation of ESCs-derived DA neurons has been demonstrated to be successful in animal models [12, 14, 15]. However, the procedure is not cost-effective, due to multiple complicated steps to drive the terminal differentiation of the cells. Moreover, tumor formation and uncontrolled cell proliferation are major issues to address before clinical applications can be realized. Tumorigenesis can be reduced by prolonged terminal differentiation and cell sorting. In one report, mitomycin treatment of ESCs has been found to increase the efficacy of DA neurons and to restore motor function without tumor formation for as long as fifteen months in mouse model [14]. Despite behavioral recovery after transplantation of ESCs-derived neural cells in animal models, little is known about the mechanisms underlying graft function. Novel technologies have been developed to dissect the mechanisms [16]. Particularly, optogenetics is harnessed to observe the graft neuronal activity and dopamine release [17].

## 3. Mesenchymal Stem Cells (MSCs)

MSCs are multipotent, nonhematopoietic stem cells which adhere to the flask surface. The cells express specific surface antigens such as CD73, CD90, and CD105 and are negative for CD45, CD34, and CD14 or CD11b and CD79a or CD19 and HLA-II [18]. MSCs are less immunological than other adult stem cells, due to the lack of MHC-II [19]. The cells can be easily isolated from bone marrow, cord blood, placenta, adipose tissue, and many other tissues. The feasibility of isolation has turned MSCs into most used cell types for stem cell therapy. However, a major obstacle in the clinical application of MSCs is their poor viability at the site of transplantation due to the harsh microenvironment that leads to cell anoikis. Various strategies can be used to improve the cell adhesion and survival of the transplanted MSCs, including pretreatment with growth factors or cytokines, hypoxic preconditioning, and genetic modifications to induce the overexpression of adhesion molecules or antiapoptotic signals [20, 21].

Human umbilical cord derived MSCs (hUC-MSCs) can be isolated with a noninvasive procedure. Although hUC-MSCs are an allogeneic cell source for recipient patients, the cells show very low immunogenicity and cannot provoke allocative lymphocyte proliferation. No tumor formation has been observed in the transplantation of hUC-MSCs into animals or humans [22]. In one report, hUC-MSCs were differentiated into DA neurons* in vitro* and then transplanted into the striatum of Parkinsonian rats. The results showed that the transplantation of terminally differentiated cells partially corrected the lesion-induced amphetamine-evoked rotation [23]. The mechanism study showed that Lmx1*α* and neurturin play an important role in the differentiation and are neuroprotective to DA neurons. When the neurturin gene was transfected into hUC-MSCs by recombinant adenovirus, the secretion of neurturin maintained the survival of rat fetal midbrain DA neurons* in vitro* and the expression of neuron-specific markers in differentiated hUC-MSCs, including tyrosine hydroxylase, *β*-tubulin III, NSE, nestin, and MAP-2. In addition, symptoms of PD monkeys were improved after cell transplantation, and there were donor neuronal-like cells which survived in the brain, observed by immunohistochemistry [24]. To further trace the donor cells, hUC-MSCs were labeled with multimodal iron oxide nanoparticles conjugated with rhodamine-B (MION-Rh). After transplantation into the striatum of PD rats, MRI was used to visualize the labeled cells. It was reported that in a neurodegenerative disease model, about 5 × 10^5^ cells could be efficiently tracked by MRI in a short term following infusion [25].

Human bone marrow derived MSCs (hBM-MSCs) are primordial cells of mesodermal origin that can be differentiated under appropriate conditions into osteoblast, chondrocyte, adipocyte, skeletal myocyte, and neuron. However, it has been demonstrated that the intravenous administration of BM-MSCs into a PD rat model is ineffective to prevent neurodegeneration. Alternatively, intrastriatal grafting is more efficient, with higher cell retention both in the substantia nigra compacta and in striatum leading to improved behavior [26]. Since the survival and differentiation rate of BM-MSCs* in vivo* is relatively low, neurotrophic factors, such as glial cell line-derived neurotrophic factor (GDNF) family ligand persephin was used to increase the survival rate of transplanted BM-MSCs, enhance the differentiation of cells into neuron- and glial-like cells, increase the content of DA in striatum, and ultimately improve the rotational behavior of PD rats [27]. Other neurotrophic factors or cytokines, such as basic fibroblast growth factor and cAMP response element binding protein (CREB), have been used to enhance the neurodifferentiation capacity of BM-MSCs and therapeutic effect in PD rats [28, 29]. Moreover, BM-MSCs could be a favorable autologous stem cell source.

Human endometrium-derived stem cells (hEDSCs) represent a new cell source for PD treatment, which is abundant and can be easily obtained by a simple, safe, and painless procedure such as Pap smears [30]. The cells are positive for CD90, CD105, OCT4, and CD44 and negative for CD31, CD34, and CD133 [31]. hEDSCs have been differentiated into DA neuron-like cells* in vitro,* which exhibited dendritic-like and axon-like projections and expressed neural cell markers including nestin and tyrosine hydroxylase [32]. After injection into the striatum of MPTP exposed monkeys, the cells can engraft into the striatum, migrate to the substantia nigra, and spontaneously differentiate* in vivo.* Increased tyrosine hydroxylase positive cells on the transplanted side and increased dopamine metabolite concentrations were also observed [33].

## 4. Neural Stem Cells (NSCs)

NSCs are not only present in the embryonic brain, but also in the adult subventricular zone (SVZ) of the lateral ventricle and subgranular zone (SGZ) of the hippocampus. NSCs can give rise to multiple types of neurons and glial cells during embryonic brain development as well as during adult neurogenesis [34, 35]. These features make NSCs a favorable endogenous cell source for neuronal replacement therapy for PD. Moreover, studies focusing on NSCs in PD models offer an earlier diagnosis and significant therapeutic effect that must replace lost DA neurons and modify the local host microenvironment to make it more conducive to the survival and differentiation of grafted DA neurons [36, 37]. Recently, NSCs have been obtained from the adult olfactory bulb (OB-NSCs), providing an attractive route that would alleviate ethical concerns associated with the use of embryonic tissues and preclude the need for invasive brain surgery [38]. Human OB-NSCs expressing neural growth factor can restore degenerated DA neurons in the striatum and ameliorate the cognitive deficiencies in 6-OHAD PD rat model [39]. In one report, mesencephalic NSCs labeled by the contrast enhancer superparamagnetic iron oxide were injected into striatum of 6-OHDA PD rats. Transplanted cells lasted for at least eight weeks after injection, detected by T2-weighted magnetic resonance imaging. Moreover, the results demonstrated that coinjection of fetal DA neurons with mesencephalic NSCs overexpressing GDNF improved functional integration of transplanted cells [40]. Human fetal NSCs (hNSCs) overexpressing GDNF could enhance survival and extensive fiber outgrowth testified at eleven months after transplantation in the midbrain of dopamine-depleted monkeys. Meanwhile, it was demonstrated that hNSCs overexpressing GDNF could direct axonal outgrowth toward an appropriate target [41]. When implanted into adult MPTP-lesioned parkinsonian monkeys, undifferentiated hNSCs spontaneously migrated from the site of transplantation to and along the impaired nigrostriatal pathway [42]. According to recent research, transplantation of NSCs could lead to reconstruction of some portion of the nigrostriatal pathway and serve as a valuable clinical tool for the parkinsonian patients.

## 5. Conclusions

**Figure 1:**
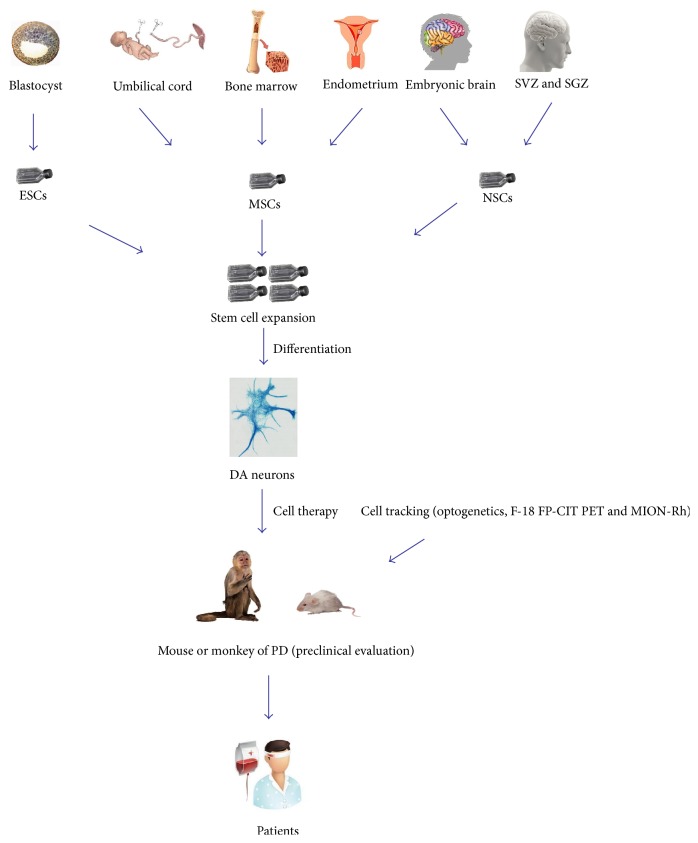
The therapeutic potential of stem cells in Parkinson's disease. EMCs, MSCs, and hNSCs from different types of organizations transplanted into a mouse or monkey model of PD as preclinical evaluation.

The field of PD stem cell therapy is rapidly evolving and approaching translational stage of clinical practice. Stem cell therapy strategies provide some potential opportunities for PD treatment, which cannot be achieved by conventional pharmacological or surgical interventions (Figure 1). However, most of the evidences regarding stem cell behavior and the potential to PD treatment have been accumulated using mouse models. Small animal models have significant limitations for preclinical validation. Recently, hUC-MSCs, hEDSCs, and hNSCs transplantation into a monkey model of PD has been reported [24, 33, 41]. Nonhuman primates have very similar central nervous systems as human, with comparable physiological parameters, pathology, and behavior in experimental models of PD. The animals have a longer lifespan which would be beneficial to perform longitudinal studies critical for most stem cell applications. Moreover, nonhuman primate models can be extrapolated readily to the dosages of biologics, the route of administration, and treatment outcomes. Therefore nonhuman primate models can bridge the gap between rodent models and humans to establish the safety of preclinical applications [43].

Previous researches have demonstrated that stem cell treatment can enhance* in vivo* neuroprotective mechanisms and induce neuroregeneration in PD models. Nonetheless, little is known about the exact mechanisms underlying graft function. It is important to develop an effective cell tracking method to noninvasively detect the infused stem cells for prolonged periods of time. The tracer material should be nontoxic and will not cause cell cycle arrest or changes in cell phenotype. The ideal material is visible under detecting instruments without the requirement for invasive methods [44, 45]. Some researchers are exploring the utility of optogenetics, F-18 FP-CIT PET, and MION-Rh to measure the therapeutic effect of stem cells in PD [17, 25, 46]. These technologies can integrate the transplantation, cell viability, animal behavior, and histopathological evaluation to enable the identification of mechanisms that drive functional recovery.

Different stem cell types for PD therapy have their strengths and weaknesses. Mouse, primate, and human ESCs have been used to differentiate into NSCs and DA neurons. However, concerns remain around the potential for risk of teratoma formation. In addition, it is often required to supplement DA neurons or DA-committed cells in the cultures to improve survival rate. Human MSCs are easy to isolate from the umbilical cord blood, bone marrow, and endometrium. MSCs can be expanded over a long period of time without ethical or technical problems. Some research centers have started clinical trials for the efficacy evaluation of human MSCs. However, the variation in cell preparation and characterization warrants further studies. NSCs regarding the potential risks have not yet been answered, which include separation and cultivation* in vitro*, their differentiation status and proliferation capacity, and long-term survival of engrafted cells.

Although these stem cell based therapies have some effects on controlling symptoms of PD, the evaluation of risk factors and potential risks must be a prerequisite step before broader clinical applications. The safety is of highest concern for any therapeutic modality. For the development of a clinically useful cell therapy in PD treatment, criteria of patient selection and graft placement should include (1) injection site, (2) the number of transplanted cells, (3) the differentiation status and proliferation capacity of transplanted cells, (4) the intended location of transplanted cells, (5) unwanted immune responses, (6) the transmission of adventitious agents, (7) tumor formation, and (8) long-term efficacy.
